# Deciphering Mode of Action of Functionally Important Regions in the Intrinsically Disordered Paxillin (Residues 1-313) Using Its Interaction with FAT (Focal Adhesion Targeting Domain of Focal Adhesion Kinase)

**DOI:** 10.1371/journal.pone.0150153

**Published:** 2016-02-29

**Authors:** Muniasamy Neerathilingam, Sneha G. Bairy, Sumukh Mysore

**Affiliations:** 1 Protein Technology Core, Centre for Cellular and Molecular Platforms (C-CAMP), NCBS-TIFR, GKVK Campus, Bellary Road, Bangalore, 560065, India; 2 Department of Biochemistry, University of Oxford, South Parks Road, Oxford, OX13QU, United Kingdom; University of Minnesota, UNITED STATES

## Abstract

Intrinsically disordered proteins (IDPs) play a major role in various cellular functions ranging from transcription to cell migration. Mutations/modifications in such IDPs are shown to be associated with various diseases. Current strategies to study the mode of action and regulatory mechanisms of disordered proteins at the structural level are time consuming and challenging. Therefore, using simple and swift strategies for identifying functionally important regions in unstructured segments and understanding their underlying mechanisms is critical for many applications. Here we propose a simple strategy that employs dissection of human paxillin (residues 1–313) that comprises intrinsically disordered regions, followed by its interaction study using FAT (Focal adhesion targeting domain of focal adhesion kinase) as its binding partner to retrace structural behavior. Our findings show that the paxillin interaction with FAT exhibits a masking and unmasking effect by a putative intra-molecular regulatory region. This phenomenon suggests how cancer associated mutations in paxillin affect its interactions with Focal Adhesion Kinase (FAK). The strategy could be used to decipher the mode of regulations and identify functionally relevant constructs for other studies.

## Introduction

**Fig 1:**
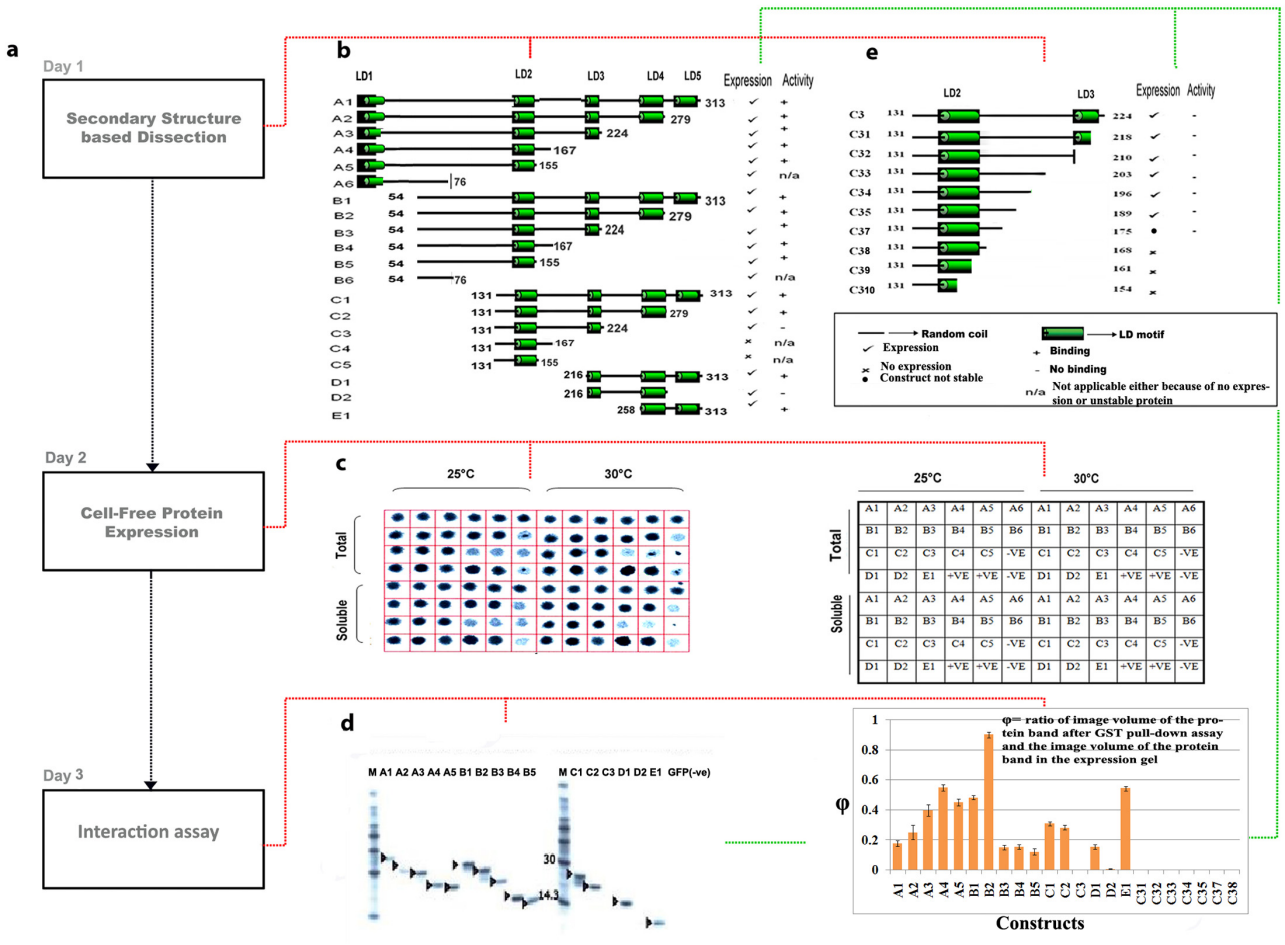
Dissection of paxillin constructs (residues 1–313) followed by expression and interaction studies. **(a)** Timeline for overall-strategy. **(b)** Illustration of solubility and activity level of linear dissected human paxillin (residues 1–313). **(c)** Phosphor screen image of filter assay for optimization of temperature for paxillin constructs (left). Tabular representation of paxillin constructs, negative and positive controls corresponding to each well in filter assay [1]. **(d)** Phosphor screen image of 10% SDS PAGE of ^35^S labeled cell-free expressed samples after GST pull-down assay of the paxillin constructs A1–E1; The right panel shows fraction of interaction of each construct with respect to B2 (since B2 showed maximum level of interaction) **(e)** Illustration of solubility and activity of dissected C3 constructs. All experiments were performed in triplicates and averaged. To rule out non-specific interactions that might occur with GST tagged FAT, GFP that was expressed in cell-free system and a reaction without DNA template were used as negative controls.

Genomic data suggests that a large proportion of eukaryotic proteins appear to adopt disordered structures in physiological conditions [1, 2]. Mutations/modifications in such IDPs are shown to be associated with various diseases (like cancer) [3]; therefore, understanding their structural behavior is critical for various applications like drug-targeting, mapping protein interactions, deciphering mode of action and finding functional relevance. However, deciphering mode of action in IDPs has been challenging given that unstructured segments render poor chemical shift dispersions and electron density in major techniques like NMR and X-ray, respectively [4]. For example, it took almost 10 years to decipher the mode of action of Sic1, a disordered protein involved in inhibition of a cyclin-dependent kinase [5]. One way to map and study the functional regions is to make truncated constructs by dissecting the whole construct rationally. A limited number of dissection constructs are usually generated; this is due to the time-consuming and challenging process of generating soluble and functionally relevant constructs when studies are performed in-vivo and constructs are prepared and tested sequentially. Here we present a simple high throughput (HTP) screening strategy (Fig 1a), which focuses on finding functionally relevant regions in IDPs based upon its interaction with a binding partner. Close to thirty dissection constructs of the IDP were generated and studied in parallel to understand the importance and functionality of the various regions of the protein. We perform cell-free expression followed by solubility check and GST pull-down interaction study in HTP format. Though both cell-free expression and GST pull-down assay have been individually performed in HTP format [6, 7], we did not find previous studies that combine the two methods in HTP format. Although the nature of interaction of IDPs with respective binding partners may vary, our strategy may be used to derive crucial insights into “structural behavior” of the unstructured segments in modulating the interaction. The strategy can also be used to identify functionally important regions in the IDP that would be suitable for further structural studies.

Disorder/Intrinsic disorder seems to be a common feature of hub proteins in eukaryotes [2], thus highlighting the need for studying the mode of action of unstructured segments in such proteins. Here we used paxillin (residues 1–313), an intrinsically disordered construct, for demonstrating this approach. Paxillin (residues 1–313) consists of multiple protein interaction sites that are connected by flexible disordered sequences [8]. The disordered regions in paxillin have been detrimental in efforts to study the complete structure of the protein due to the demerits mentioned previously. This explains the lack of structural details of regulation of paxillin binding. Residues 1–313 of paxillin consist of five leucine-rich sequences LD1-LD5 (with consensus sequence: LDXLLXXL), termed LD motifs, which are highly conserved between species and other family members such as Hic-5, leupaxin and PAXB [8]. Paxillin interacts with multiple proteins involved in cell migration, actin rearrangements and cell proliferation [9]. Mutations in paxillin are shown to be associated with lung cancer [3, 10]; and the differential expression of paxillin is associated with various forms of cancer and other diseases such as Alzheimer’s and inflammation [11–13]. This implies the importance of studying the structural and functional characteristics of paxillin. Most paxillin studies focus on interactions of LD motifs with proteins such as focal adhesion kinase (FAK), vinculin and v-crk, providing clues towards their importance in deciphering the functionality of paxillin [8, 14, 15]. Though regions of paxillin that bind to various partners were deciphered through previous studies, the basis of effect of mutations in paxillin on binding its partners was not explained. Mutations in paxillin, some that were observed to be associated with cancer were positioned in the intrinsically disordered regions between the LD motifs and not on the motifs themselves [3, 10]. For example, P30S, G105A and A127T mutations lie between LD1 and LD2 motif; P233L and T255I mutations lie between LD3 and LD4 motifs. This shows that the LD motifs alone do not govern the functionality, but unstructured regions linking the LD motifs could play a major role. In normal conditions, FAT (Focal adhesion targeting domain of FAK) binds hydrophobically through its HP1 (Hydrophobic patch 1) and HP2 (Hydrophobic patch 2) sites to paxillin LD motifs—LD2 and LD4 [16, 17], which lead to activation of binding sites for other proteins on paxillin. LD2 preferentially binds to the HP2 site, whereas LD4 preferentially binds to the HP1 site [18]. In a state of cancer caused by mutations in paxillin, the LD interactions could be hindered, as mutations in the unstructured segments result in abnormal binding of FAK to either of the LD motifs [9]. Here we wanted to locate the region involved in the structural modulation of paxillin-FAT interaction by adopting a simple approach (Fig 1) that involves dissected proteins generated using cell-free protein expression coupled with protein-protein interaction study. We map the disordered proteins’ structural importance to understand the function and modulation of paxillin-FAT interaction in days rather than months (Fig 1a).

## Results

### Dissection and identification of fragments of paxillin (residues 1–313) with functional relevance

**Fig 2:**
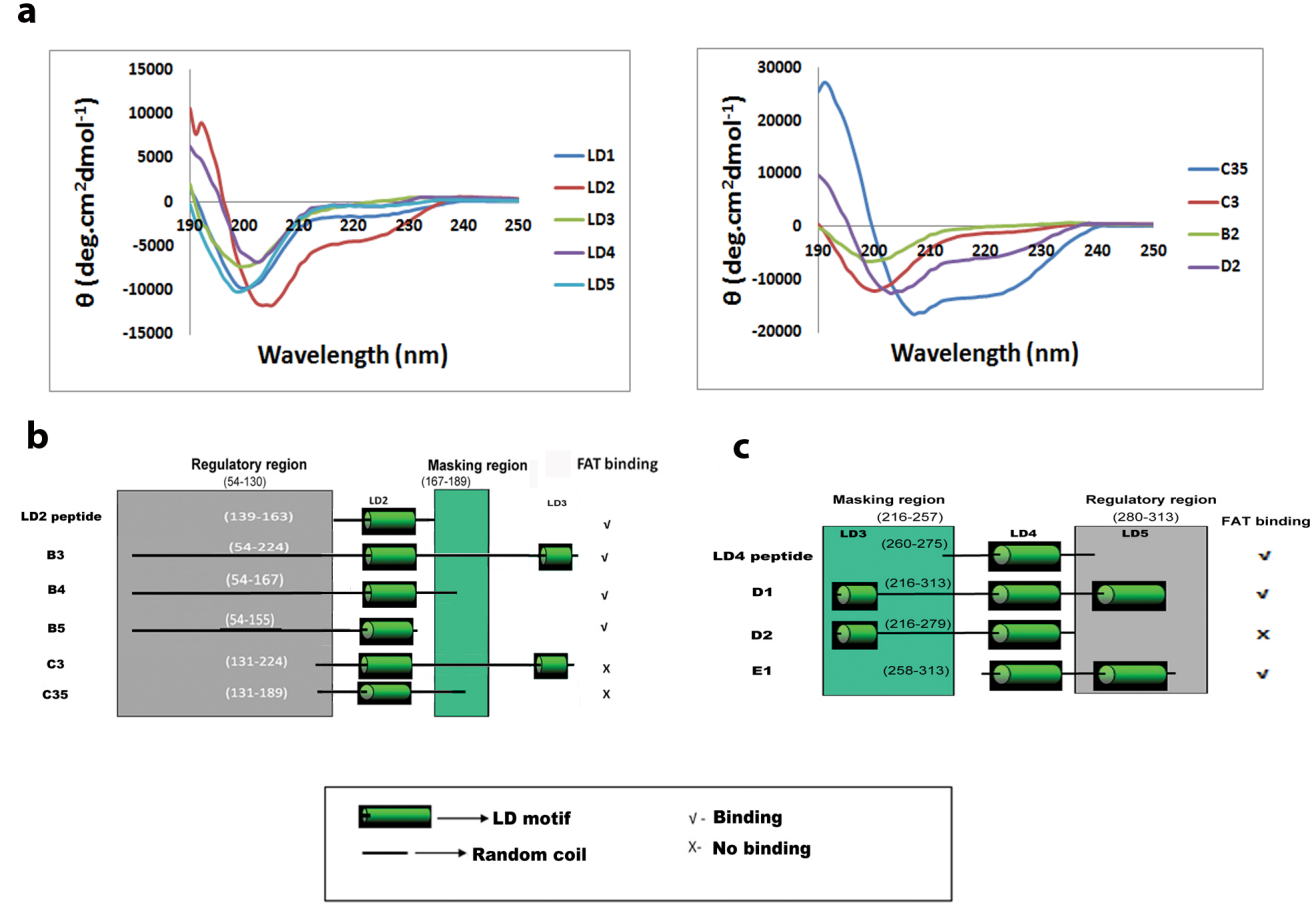
Regulatory and masking regions around paxillin’s LD2 and LD4 and their circular dichroism spectra. (a) CD spectra of paxillin LD peptides (LD1-LD5) and constructs: B2, C3, C35 and D2. CD spectra of LD2, LD4, C35 and D2 constructs showed negative bands at 222nm and 206nm and a positive band at 192nm that confirms the presence of alpha helical content thus may behave as folded effector binding sites. However, LD1, LD3, LD5, B2 and C3 do not show the characteristic peaks of secondary structures, thus may behave as unfolded effector binding sites. (b) LD2 regulatory region (54–130) and masking region (167–224) evidenced by constructs B3, B4 and B5. (c) LD4 regulatory region (216–257) and masking region (280–313) evidenced by constructs D1, D2 and E1.

We dissected paxillin (residues 1–313) (Fig 1b) into nested sets using PCR such that each of the constructs had either or both LD2 and LD4 motifs (S1 Fig and S1 Table). Further, these constructs were expressed in soluble form using small-scale cell-free expression system in a 96 well format (Fig 1c). However, all constructs except A6, B6, C4 and C5 expressed detectable amounts of protein (S2a Fig and S2 Table). The failure in expression of the above constructs could be due to the instability of the smaller peptide fragments that might be susceptible to proteolytic cleavage [19]. Soluble protein from small-scale expression of the dissected constructs namely A1, A2, A3, A4, A5, B1, B2, B3, B4, B5, C1, C2, C3, D1, D2, and E1 were pulled down and analysed (Fig 1d). Although constructs A1–A5, B1–B5, C1, C2, D1 and E1 interacted successfully, C3 (containing LD2) and D2 (containing LD4) failed to interact (Fig 2c) despite containing LD motifs. However, based on previous reports [8, 16, 17], we expected all constructs containing either LD2 and/or LD4 to interact with the FAT domain. Therefore, this led us to suspect that intra-molecular auto-inhibition in unstructured segments modulated binding of FAT to LD motifs in paxillin.

### Identification of regulatory regions and their mechanisms

**Fig 3:**
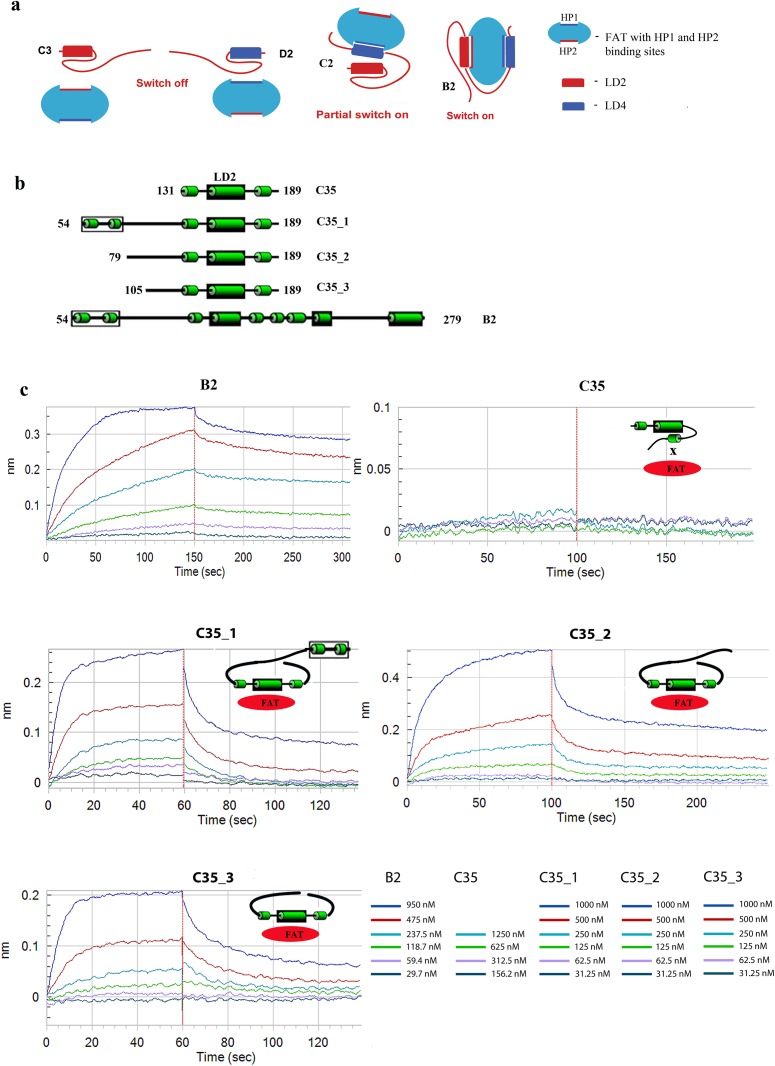
Binding studies of paxillin constructs using Bio-layer Interferometry on OctetRed96. (a) Switch off in C3 and D2 on LD2 and LD4 respectively; Hypothesis of partial switch on when regulatory region of LD2 is absent, as evidenced in C2. (b) Concentration calibration curves depicting binding of constructs B2, C35, ‘54–189’, ‘79–189’, ‘105–189’ with GST-FAT. The data is representative of a single experiment. Each experiment was performed at-least thrice. (c) Illustrations of C35, C35_1, C35_2 and C35_3.

To investigate the non-interaction of C3, a series of C3 deleted constructs (C31 –C310) (Fig 1e, S1 Table) were generated to determine the internal region that influenced the non-functioning of C3. C36 linear template could not be amplified for expression. As solubility of C3 could play a critical role in determining interaction, the homogeneity of the sample was confirmed by capillary electrophoresis under non-reducing conditions [20] (See S3 Fig). The linear templates—C31, C32, C33, C34 and C35 were successfully expressed in soluble form, The other C3 deleted constructs did not express due to issues related to small size as described earlier. Surprisingly, none of the C3 deleted constructs interacted with FAT despite the presence of the LD2 motif, although constructs such as B3, B4 and B5 that contain regions overlapping with C3 showed interaction (S2b and S2c Fig, Fig 1d and 1e). Here B3 that included the whole of C3 and unstructured segment 54–130 showed interaction (Fig 1b). Constructs B4 and B5 also containing residues 54–130 showed interaction despite differing from B3 by lacking regions 167–224 and 155–224, respectively. Interestingly, the non-interacting constructs C3 and C35 do not contain 54–130 residues, but include the regions 167–224 and 167–189, respectively (Fig 1b). Here constructs containing region 167–189 but lacking 54–130 did not interact with FAT despite LD motif alone showing interaction (switch off) (Fig 3a). Whereas, if 54–130 was included, interaction was reinstated (switch on) (Fig 3a). This clearly shows that interaction of LD2 in construct C35 is masked by residues 167–189 (masking region) (Fig 2b). The constructs B3 and B4 binding to FAT despite the presence of the masking region led us to conclude that the region 54–130 (regulatory region) acts to remove the masking effect (Fig 2b).

**Fig 4:**
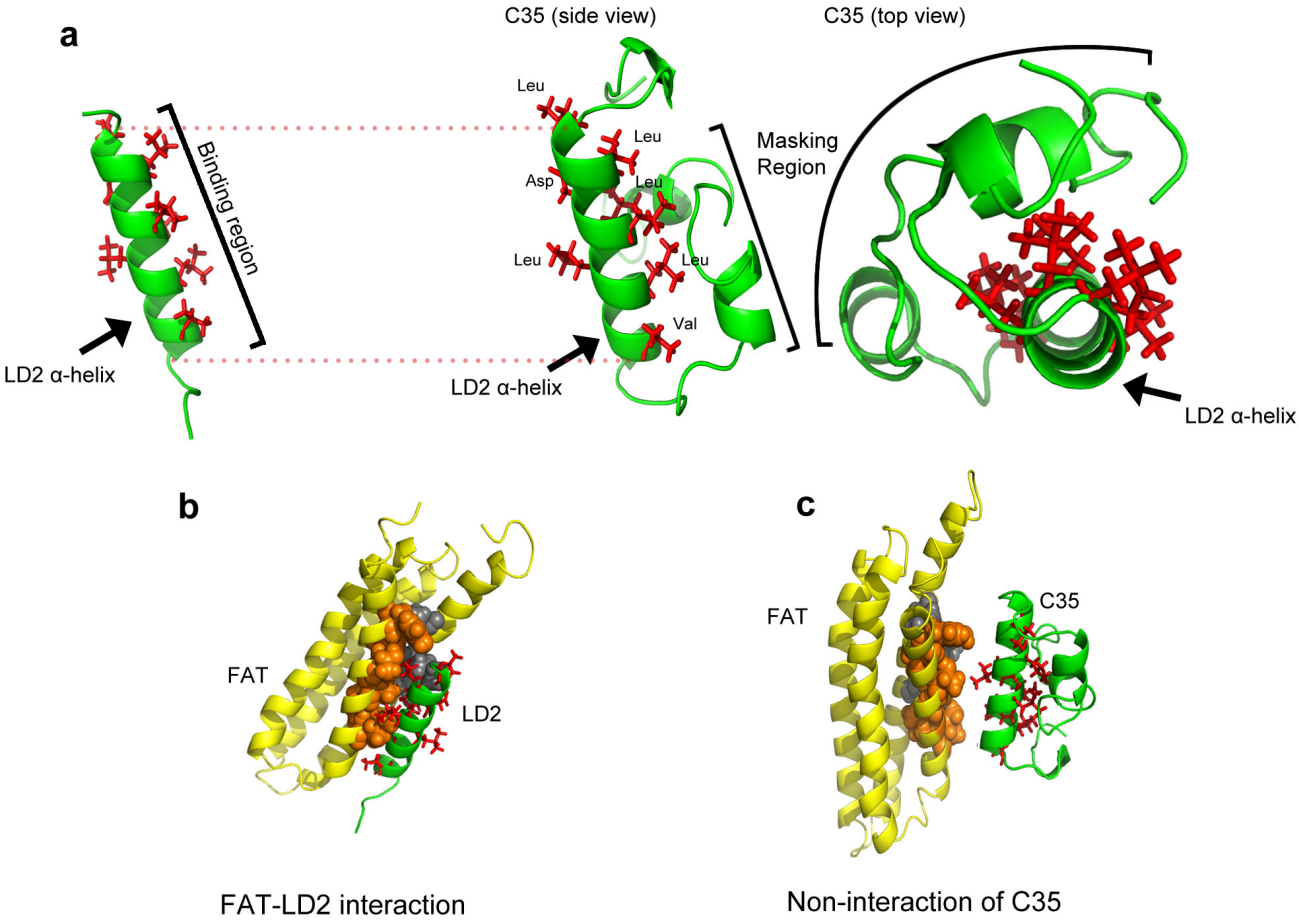
*In-silico* analysis of non-binding of C35. (a) LD2 crystal structure from PDB id: 1K05 (left) being compared with the LD2 structure in the side view and top view of C35 structure showing the masking of the hydrophobic binding region predicted through HMM based SAM-T08 software. The LD2 binding region and the masking regions are depicted by the bracketed region. (b) Docking control showing FAT (co-ordinates from PDB id: 1K05) and LD2 (co-ordinates from PDB id: 2L6F, NMR model # 1) interaction using Hex 6.3 software. (c) Docking of C35 with FAT showing non-interaction due to masking effect. The sidechains of the active residues are shown as red sticks. The hydrophobic patch—HP2 in FAT molecule, which preferentially binds to LD2 is shown as a space filling model in orange (part of helix 1 of FAT) and grey (part of helix 4 of FAT) colors.

Similar to LD2, LD4 in construct D2 containing 216–257 (masking region) requires additional residues of paxillin 280–313 (regulatory region) for FAT binding (Fig 2c), which was demonstrated by showing the interaction with constructs D1 (spanning region 216–313) (Fig 1b) and E1 (spanning region 258–313). To visualize the non-binding of FAT to C35, in-silico methods were employed to model the C35 construct and docked with the crystal structure of FAT (1K05, residues 916–1050 [21]) (Fig 4). The docking results showed a clear masking effect in the C35 construct by the 167–189 (masking region) residues. The constructs B2, C3 and C35 were also structurally characterized using CD analysis (Large scale cell-free expression was performed for this purpose, see S3 Fig). The percentage of alpha helical content was found to be much higher in C35 (95.32%) as compared to B2 (12.43%) (Fig 2a, S3 Table). Therefore, the dissection(s) of B2 to C35 allowed the identification of structured regions (C35) as compared to the disordered B2. Further, it showed that the LD2 peptide and C35 have significant alpha-helical structures that do not translate into functional similarity as evidenced by the inability of C3, C35 and D2 to bind to FAT. Moreover, LD2 peptide binds to FAT while C35 does not (Fig 1e and S2b and S2c Fig). A similar observation was made when comparing the ability of LD4 peptide and the inability of D2 to bind to FAT despite both having detectable α-helical content (Fig 1b). Thus, these results confirm the existence of masking and regulatory regions (Fig 2b and 2c) that determine switch on and off and in turn, intra-molecular auto-inhibition. C2 showed activity despite missing regulatory regions for both LD2 and LD4 (similar activity observed in C1). This could be because the unfolded nature of LD3 effector binding site that is located between LD2 and LD4 is flexible to mask only a single LD motif but not both (Partial switch on, Fig 3a).

To predict the influence of this structural modulation, the state of LD motifs structurally before and after binding to FAT had to be understood. CD spectra of LD1, LD3 and LD5 peptides showed characteristics of random coil (Fig 2a, S3 Table) thus validating that the LD1, LD3 and LD5 motifs could exist as unfolded effector binding sites (not available for interaction) in our study and could fold upon undergoing allosteric changes after binding to their respective targets.

### Validation of protein-protein interaction study using bio-layer interferometry studies

Bio-layer interferometry studies were performed to further validate the interaction studies and also to get insights into the binding affinities. Here apart from constructs B2 and C35, three other constructs that include different lengths of the regulatory region along with the C35 region were used for the studies, namely—Construct C35_1(54–189); Construct C35_2 (79–189) and Construct C35_3 (105–189) (See Fig 3b and S4 Fig). As seen in Fig 3c and Table 1, B2 shows maximum binding with K_D_ value in the nano-molar range and the curves fit into a 1:1 binding model. C35 shows negligible binding and the rest of the constructs show binding lower than B2 with K_D_ values in micro-molar range and the curves fit into a 2:1 binding model (See S5 Fig).

**Table 1:** K_D_ values and curve fitting details of B2, C35, C35_1, C35_2 and C35_3 using Bio layer interferometry.

Sl No	Construct	K_D_ (M)	k_on_(1/Ms)	k_off_(1/s)	Full X^2	Full R^2
1	B2	3.61E-08	4.75E+04	1.71E-03	0.129692	0.994738
2	C35	Nil	Nil	Nil		
3	C35_1	5.39E-06	2.25E+04	1.21E-01	0.025607	0.993133
4	C35_2	1.69E-05	9.71E+03	1.64E-01	0.033629	0.998299
5	C35_3	1.45E-06	7.29E+04	1.06E-01	0.007434	0.995805

The values were calculated as mentioned in the methods section. K_D_ refers to the dissociation constant, k_on_ refers to association rate, k_off_ refers to dissociation rate, full X^2 refers to the sum of squared deviations, which provides a measure of error between the fitted line and the experimental data; and full R^2 indicates how well the experimental data and the fit correlate.

## Discussion

According to previous reports, FAK has to bind to both LD2 and LD4, failing which phosphorylation during signalling is reduced [8], which is observed in case of cancer [3], thus resulting in abnormal functioning of paxillin. We investigated this by analysing B2, which showed higher interaction than B1, despite missing the regulatory region of LD4 (Fig 1b). Similarly, C2 showed activity despite missing regulatory regions for both LD2 and LD4 and the presence of masking regions (similar activity observed in C1). This suggests that the masking region that is located between LD2 and LD4 is flexible to mask only a single LD motif but not both (Fig 3a). Interestingly, paxillin mutations associated with lung cancer were observed in the unstructured segments, particularly the regulatory region of LD2 and masking region of LD4 [3]. We hypothesize that these mutations prevent proper functioning of the regulatory regions, thus resulting in masking of either of the LD motifs causing abnormal functioning of paxillin. Evidence that these regions regulate FAT-paxillin binding was further provided in our study in the form of the bio-layer interferometry results; where C35 did not show any binding, but the constructs that included different lengths of the regulatory region along with the C35 region showed binding with K_D_ values in the micro-molar range. This suggests that the LD2 region in these constructs is not masked, since it is seen in previous studies that the K_D_ value for FAT binding to a single LD motif of paxillin is in micro-molar range. It also suggests that the region between residues 105–131 is sufficient for preventing the masking of LD2 region, thus allowing interaction with FAT (See illustrations in Fig 3b). Except B2 (that had a 1:1 binding stoichiometry and higher binding affinity), all other constructs (C35_1, C35_2, C35_3) showed a 2:1 binding stoichiometry. This suggests that both LD motifs of B2 engage both the FAT HP sites thus resulting in higher affinity; whereas in the other 3 constructs (C35_1, C35_2, C35_3), each FAT HP site (HP1 and HP2) interacts with individual molecules thus giving a 2:1 stoichiometry. This is in agreement with previous studies where both the LD motifs were found to interact with both HP1 and HP2 hydrophobic patches of FAT [16]. The higher affinity of B2 to FAT could be due to presence of both LD2 and LD4; the proposed intra-molecular regulatory regions could also play a role in the increased affinity. Therefore, we understand that the abnormal modulation in cancer involves redirection of FAK to a single LD motif; and targeting drugs for re-establishing the function at regulatory regions could be critical.

Unlike many existing techniques like array based yeast two hybrid assay, phage display method and tandem affinity purification; the strategy used here (combination of cell-free expression, filter based solubility assay and interaction study in HTP format) facilitated quick identification of the role of unstructured regions involved in paxillin-FAT interaction in HTP format. Particularly, in paxillin-FAK interactions, which determine focal adhesion and cellular signalling, we understood the structural masking and unmasking behaviour of unstructured segments in paxillin to determine FAK interaction. The structure of paxillin is not yet elucidated due to difficulties with respect to its disordered nature. In this study, the templates that we generated using the high throughput dissection strategy allowed us to analyze various regions of paxillin, with respect to structure, solubility and function. To our knowledge, this study is the first report of switch on and off mechanisms working together in controlling allosteric modulation/auto-inhibition in a human hub protein. As many eukaryotic proteins are disordered, our study opens avenues for analyzing novel modulations at allosteric sites using appropriate interaction studies, which could lead to identification of new drug target sites. In this regard, we hope the above strategy will be instrumental in understanding mechanisms of other disordered proteins as well, in days rather than months. This strategy could also be used as an initial screening method for techniques like SAXS, smFRET and others.

## Materials and Methods

### PCR for dissecting paxillin constructs

The Advantage^®^-GC cDNA PCR kit from BD Biosciences (CLONTECH UK, Oxford, UK) was used to amplify the paxillin gene from a human source of cDNA (Whole Tissues- from BD Biosciences, CLONTECH UK). Linear fragments were successfully amplified from this cDNA. Via a second overlap extension PCR reaction, regulatory elements necessary for expression in a prokaryotic system, based on T7 polymerase were introduced. The primer details for generation of linear DNA templates of constructs A1–A6, B1–B6, C1–C5, D1, D2 and E1 are provided in S1 Table and the primers used to generate linear templates for the C3 deletion constructs (C31-C30) are listed in S1 Table. The following conditions were used for all other paxillin constructs (primary PCR product) in this work. The conditions of the PCR were 1 min/94°C– 1 cycle; 30 sec/ 94°C; 3:30 min/ 68°C; 25 cycles total; 3:30 min/ 68°C– 1cycle; 15°C until the program is stopped. To add other transcriptional elements into the linear templates (or into the primary PCR product) for cell-free expression, splice overlap PCR was used for generating nested sets of dissected paxillin constructs using the RTS *E*.*coli* linear template generation set (HA-tag), by following the manufacturer instructions (Roche Diagnostics).

### Preparation of cell-free extracts, protein expression and purification

Cell-free extract of BL21 codon plus RIL was prepared and protein expression was performed according to Kigawa *et al*.’s procedure [22]. The protein expression level was determined based on the normalization of the methionines present in the constructs (S4 Table).

#### Small-scale cell-free expression using laboratory reagents

The complete total reaction mix was 30 μl, which contained 55 mM Hepes-KOH pH 7.5, 4% polyethylene glycol (PEG) 8000, 210 mM potassium glutamate, 1.8 mM DTT, 1.2 mM ATP, 0.8 mM each of CTP, GTP, UTP, 0.64 mM 3’, 5’-cyclic AMP, 35 μg/ml folinic acid, 27.5 mM ammonium acetate, 80 mM creatine phosphate, 0.25 mg/ml creatine kinase, 175 μg/ml *Escherichia coli* total tRNA, 0.05% sodium azide, 10.7mM magnesium acetate, 1 mM each amino acid, 0.5 mM methionine, 0.5μl of ^35^S labelled methionine (1 mCi/ml)–from Amersham Bioscience, Little Chalfont, UK, 0.27 μl T7 RNA polymerase (200 U/μl, Ambion, Huntingdon, UK), 7.2 μl S30 extract and 60 to 250 ng of DNA template. The reactions were incubated within a polypropylene 96-well plate (Anachem, Luton, UK) in a Dyad DNA Engine thermo cycler (MJ Instruments, UK) for 90 minutes.

#### Large-scale cell-free expression

For large-scale expression, 3 ml of the warmed reaction mix with unlabelled amino acids was placed in a dialysis bag (Spectra/Por 2.1, 50 kDa MWCO -Spectrum Labs, Dealer: NBS Biological Ltd., Cambridge, UK) along with ~15 μg plasmid DNA. 30 ml of external solution/ or feeding solution was prepared, consisting of the same composition as the reaction mix except for the creatine kinase, the plasmid DNA, the T7 RNA polymerase, the S30 extract and also containing an additional 4.2 mM magnesium acetate. The reaction was incubated at 30°C or optimised temperature at 160 rpm for 12 hours.

#### HTP solubility filter assay

We used 96 well format HTP assay to check protein solubility. 2 μl of the reaction mixture was labelled with ^35^S and applied to a Type GF/C glass fibre filter using multi-channel pipette before and after centrifugation at 6,500 g for 30 minutes. The proteins were then precipitated and the free amino acids were removed by a 10 minute wash with TCA (10%, w/v) in sodium pyrophosphate (1%, w/v) and washed twice for 5 minutes each with TCA (5%, w/v) ^14^. The dried filters were exposed for 10–20 minutes to 20 by 25cm general purpose phosphor screens which were subsequently read with a Storm 820 phosphorimager and the images processed with Image Quant software.

#### Protein purification

The large scale cell-free reaction mixture from the dialysis bag was buffer exchanged to 50 mM Tris, pH7.5, 200 mM NaCl with a Centricon ultrafree concentrator MWCO 5–10 kDa (Millipore (UK) Ltd, Watford, UK) and applied to a HisTrap 1 ml column (Amersham Biosciences, UK) equilibrated with the same buffer. The His-tagged protein was eluted with an imidazole gradient (0 to 0.5 M) in the same buffer. Construct B2 was further purified using HPLC since more than a single band was seen for the purified protein. Cleared supernatant was acidified to pH = 3–4 with TFA prior to injection onto the HPLC column. A C4 reverse-phase column (Size: 250x 10.00 mm, micron, Phenomenax, Macclesfield, UK) was used for the purification on a Varian HPLC system. All elutions were performed (flow rate = 3 ml/min) using a gradient of 2% B to 80% B over 30–40 minutes where B is 80% acetonitrile, 0.1% TFA.

### Glutathione S—Transferase (GST)-Pull down assay

A fusion protein of GST with the FAT (focal adhesion targeting) domain of FAK was kindly provided by Dr. M. K. Hoellerer (Department of Biochemistry, University of Oxford). In brief, recombinant human FAK_892–1052_ (FAT) was cloned into pGEX4T1 vector and expressed in E.coli BL21 (DE3) followed by purification using GST-4B beads. The same construct was also synthesized through GeneArt^™^ (Gene synthesis and cloning services offered by ThermoFisher Scientific) and cloned into pGEX4T1. The same procedure mentioned above was followed to obtain the GST-FAT fusion for bio-layer interferometry studies.

It was well characterized that GFP would not bind to GST-FAT. Hence, it was used as negative control. However, to make sure that GST does not interact with the constructs, GST was made to interact with one of the constructs, A3, that showed non-interaction S2d Fig).

GST-4B beads were diluted with 1x assay buffer (20 mM Tris pH 8.0, 0.15 M NaCl, 0.05% Tween x100) to obtain 50% or 2.5 μg/μl of slurry. The total pull-down assay working volume of 231 μl contains 20 μl GST-4B bead slurry, 1 μl of GST-FAT (17 μg/μl), 10 μl of ^35^S labelled sample of paxillin, and 200 μl of assay buffer (1x). The various regions of ^35^S methionine labelled paxillin constructs were expressed and incubated with GST-FAT (45 kDa) and GST-4B beads (Amersham Biosciences, UK) in a 96 well filter plate for 2 hours in the cold room (4°C to 6°C). In order to remove the unbound paxillin constructs, the beads were washed 4 times with assay buffer (20 mM Tris pH 8.0, 0.15 M NaCl, 0.05% Tween x100) on a vacuum manifold and processed for image analysis. The bound protein was eluted in boiling sample buffer and visualized by phosphor image analysis after running the samples on a 10% SDS PAGE gel (The SDS PAGE was exposed for 10–20 minutes to 20 by 25cm general purpose phosphor screens which were subsequently read with a Storm 820 phosphorimager and the images processed with Image Quant software). The degree of interaction between various constructs of paxillin and FAT were calculated by dividing the image volume of a band in SDS-PAGE that corresponds to the construct after GST pull-down assay by the image volume of a band in SDS-PAGE that corresponds to the construct expression. The image volumes of bands in SDS-PAGE gel were normalised with the number of methionines in the constructs (S4 Table).

### Bioanalyser (Agilent Technologies)

The purified protein homogeneity was determined and protein concentration estimated by capillary electrophoresis under non-reducing conditions on a 2100 Bioanalyzer using the Protein 50 Kit (Agilent Technologies).

### Biophysical studies

#### Mass spectroscopy

The purified protein molecular weight (MW) was determined by electron-spray ionization mass spectroscopy on a VG Platform II ESI-MS (S4e and S4f Fig).

#### Circular dichroism

The analysis was performed on a Jasco J-720 spectro-polarimeter with the temperature of the circulating water bath maintained at 25°C. The wavelength scan parameters were set as follows: start wavelength: 250 nm, end wavelength: 190 nm, step resolution: 1 nm, speed: 50 nm/min, accumulation: 16, response: 1, bandwidth: 1 nm and sensitivity: 20 mdeg. The spectra were plotted in Excel from text files of data points averaged from the 16 scans. The overall shape of the spectra plus wavelength positions of maximum and minima and points of inflexion were analysed in order to compare the various paxillin constructs and paxillin LD’s peptides. The LD1, LD2, LD3, LD4 and LD5 peptides were kindly provided by Dr. Maria Hoellerer (Department of Biochemistry, University of Oxford). These LD motifs were synthesized by Dr. G. Bloomberg (Department of Biochemistry, Bristol, UK) using CEM Liberty Blue automated, microwave-assisted peptide synthesizer that supports standard Fmoc solid-phase synthesis. Sequences of the five LD motifs are as follows: LD1—MDDLDALLADLESTTSHISK, (human paxillin residues 1–20); LD2- NLSELDRLLLELNAVQHNPP, (human paxillin residues 141–160); LD3 –VRPSVESLLDELESSVPSPV, (human paxillin residues 213–232); LD4—ATRELDELMASLSDFKFMAQ, (human paxillin residues 262–281); LD5 –PGSQLDSMLGSLQSDLNKLG, (human paxillin residues 296–315). All the synthesized peptides were dissolved in 10mM potassium phosphate buffer (pH 7.4) in order to get 20μg in 200μl for CD analysis. The background solution signal was subtracted and the mdeg values were converted to mean residue molar ellipticity (θ (deg.cm2dmol-1)). The *k2d3* programme was used for predicting the structural components of paxillin constructs. *k2d3* was performed via a web server (http://cbdm-01.zdv.uni-mainz.de/~andrade/cgi-bin/k2d3/k2d3_set1.pl) by submitting CD values ranging from 190nm to 240nm.

#### Bio-layer interferometry studies

GST labeled FAT along with B2, C35, C35_1, C35_2 and C35_3 were cloned (FAT was cloned into pGEX4T1 vector; other constructs were cloned into pET28a; See S1 Table), expressed in BL21DE3 strain and purified (see S4 Fig). The purified constructs were concentrated to 1mg/ml. GST-FAT was then immobilized on Anti-GST (GST) Biosensors and the binding with the above paxillin constructs was performed on the ForteBio—Octet RED96 System. The FAT Protein sample was diluted to 20 μg/ml in PBS before immobilization. All the analyte samples (the paxillin constructs) were diluted to the below concentration ranges using PBS buffer having Tween 20(0.05%) and BSA (1%).

- B2: 0.95 μM – 0.0297 μM
- C35: 5 μM – 0.15 μM
- C35_1: 1 μM – 0.031 μM
- C35_2: 1 μM – 0.031 μM
- C35_3: 1 μM – 0.031 μM

Cycles for analysis involved obtaining a 60 s baseline followed by a 60/100/150s association step and a 150s dissociation step. The assay was repeated with the reference biosensors to correct for non-specific interactions and the entire assay was repeated in triplicate. The curves obtained were then subjected to global fitting and the kinetic parameters were calculated using ForteBio software (see S5 Fig). The equations for the calculations of k_on_, k_off_ and KD are included in the Supplementary information text (S1 Text). The complete details of the equations used for fitting the curves can be obtained in the document–‘Biomolecular Binding Kinetics assays on the octet platform’, downloaded from the website—http://www.fortebio.com/literature.html.

### Structure prediction and docking

HMM based SAM-T08 [23] online server (threading based structure prediction) was used to obtain the structure of C35. FASTA format of the protein sequence is submitted for structure prediction. The model with the lowest E-value and the highest confidence score is chosen for further studies. For docking, Hex—version 6.3 executable file of the docking software was downloaded from the website http://hex.loria.fr/dist/index.php. The receptor (FAT) and the ligand (LD2 or C35) molecules both in PDB format are first uploaded into Hex. The ligand is then positioned towards the hydrophobic binding site of FAT. An initial distance of 20–25 Å between the receptor and ligand was chosen. Crystal structure 1K05 (FAT) was used as receptor for docking. The structure of LD2 (that was used as ligand) was modeled using the NMR structure of FAT bound to LD2 and LD4 motifs (PDB: 2L6F, model #1)". During docking, the rotation of receptor was maintained at 45° and that of ligand at 180°. After docking, molecular mechanics minimization was done by Hex. The docking model with minimum total energy was selected for analysis.

## Supporting Information

S1 Fig PCR dissection of paxillin constructs. (a): Analysis of primary and secondary PCR products for cell-free protein expression using 0.8% agarose gel. Lanes: M—Markers (100bp); Lanes 2, 4, 6, 8, 10, 12, 15, 17, 19, 21, 23, 25, 29, 31, 33, 35, 37, 40, 42 and 44 show the primary PCR products of constructs A1, A2, A3, A4, A5, A6, B1, B2, B3, B4, B5, B6, C1, C2, C3, C4, C5, D1, D2 and E1, respectively as shown in Fig 1a; Lanes: 1, 14, 27, 28, 39 and 46 are pIVEX2.4d containing T7 promoter (T7P) and T7 terminator (T7T); Lanes 3, 5, 7, 9, 11, 13, 16, 18, 20, 22, 24, 26, 30, 32, 34, 36, 38, 41, 43, and 45 are secondary PCR products obtained from the splicing of primary PCR products and pIVEX2.4d to incorporate the T7P and T7T for obtaining linear DNA templates of constructs A1, A2, A3, A4, A5, A6, B1, B2, B3, B4, B5, B6, C1, C2, C3, C4, C5, D1, D2 and E1 respectively; (b): Schematic of human paxillin (residues 1–313) showing oligonucleotides (forward and reverse primers) used for primary PCR to dissect the molecule.
(TIF)

S2 Fig Small scale expression of paxillin dissection constructs A1 to E1; Expression and interaction studies of C3 dissected constructs. **(a)**: Phosphor screen image of 10% SDS PAGE gel for ^35^S labeled paxillin constructs (indicated by the purple arrowhead) (left). Optimization of protein expression was performed at 25°C and 30°C with 8.33μg/ml of template DNA, here the positive control was GFP (+ve) expressed the same conditions and negative control (-ve) was cell-free extract without DNA [1]. The right panel shows the extent of solubility of each construct; **(b)**: Phosphor screen image of 10% SDS PAGE gel for ^35^S labeled, small scale expressed C3 dissected constructs. **(c)**: GST pull down assay showing B2 and E1 interaction and C3 deleted constructs (C31-C35) showing lack of interaction. **(d)**: Interaction assay of a paxillin fragment, A3 showing its non-interaction with GST.
(TIF)

S3 Fig Large scale expression and purification for CD analysis. **(a)**: 12% SDS PAGE analysis of expressed and purified B2. M-Marker. The proteins were visualized with Coomassie Brilliant Blue; **(b)**: Capillary electrophoresis (using Agilent Bioanalyser) of paxillin construct C3 under non-reducing conditions. The protein size is estimated by comparison with protein standards (6 to 53 kDa) and the sample concentration by comparison of peak area; **(c)**: Capillary electrophoresis (using Agilent Bioanalyser) of paxillin construct C35 under non-reducing conditions; **(d)**: Capillary electrophoresis (using Agilent Bioanalyser) of paxillin construct D2 under non-reducing conditions. SP: System peak; LM: Lower marker; HM: Higher marker **(e)**: The deconvoluted ESI-mass spectrum of paxillin C3 construct. The calculated MW for the major species agrees with two different molecular weights to the same species of C3. The mass difference between the two main peaks (12792Da -12634Da = 158Da) is the approximate difference expected for deletion of an amino terminal formyl methionine (159Da); **(f)**: The deconvoluted ESI-mass spectrum of paxillin C35 construct. The calculated MW (8862 Da) of the major species exactly agrees with a protein without an amino-terminal methionine, as expressed from pIVEX2.4dC35.
(TIF)

S4 Fig Expression and purification of B2, C35, C35_1, C35_2 and C35_3 for Bio-Layer Interferometry studies. All the above constructs were expressed in *E*.*coli*, BL21DE3 strain. UI refers to uninduced and M refers to Marker. GST-FAT, B2 and C35_1 were run on 12% SDS PAGE, C35_2, C35_3 and C35 were run on 15% SDS PAGE. The proteins were visualized with Coomassie Brilliant Blue. **(a)** Lane 1: Soluble fraction of expressed GST-FAT; Lane 2: Purified GST-FAT. **(b)** Lane 1: Soluble fraction of B2; Lane 2: Purified fraction of B2. **(c)** Lane 1: Soluble fraction of C35_1; Lane 2: Purified fraction of C35_1. **(d)** Lane 1: Soluble fraction of C35_2; Lane 2: Purified fraction of C35_2. **(e)** Lane 1: Soluble fraction of C35_3; Lane 2: Purified fraction of C35_3. **(f)** Lane 1: Soluble fraction of C35; Lane 2: Purified fraction of C35. **(g)** Precession plus dual color Molecular weight marker with kDa values.
(PDF)

S5 Fig Global curve fitting of the curves obtained with Bio-layer interferometry studies, where the paxillin constructs were subjected to interaction with GST-FAT bound to anti-GST biosensor. B2 shows maximum binding with K_D_ value in the nano-molar range and the curves fit into a 1:1 binding model. C35 shows negligible binding and the rest of the constructs show binding lower than B2 with K_D_ values in micro-molar range and the curves fit into a 2:1 binding model.
(PDF)

S1 Table Primers used for the generation of linear DNA templates with N-terminal His-tag of dissected constructs of paxillin (A1–A6, B1–B6, C1–C5, D1, D2 and E1). (PDF)

S2 Table Expression of paxillin constructs with the corresponding incubation temperature. (PDF)

S3 Table Alpha helix, beta helix content and random coil content of constructs B2, C3 and C35 and the LD motifs 1–5 according to CD analysis. (PDF)

S4 Table Parameters and details for the paxillin constructs. (PDF)

S1 Text Equations for the calculations of k_on_, k_off_ and K_D_. (DOCX)
