# Supplementary figures and images for: Deciphering Mode of Action of Functionally Important Regions in the Intrinsically Disordered Paxillin (Residues 1-313) Using Its Interaction with FAT (Focal Adhesion Targeting Domain of Focal Adhesion Kinase)
Source: PLoS One. 2016 Feb 29;11(2):e0150153. doi: 10.1371/journal.pone.0150153 (PMC4771712; doi:10.1371/journal.pone.0150153)

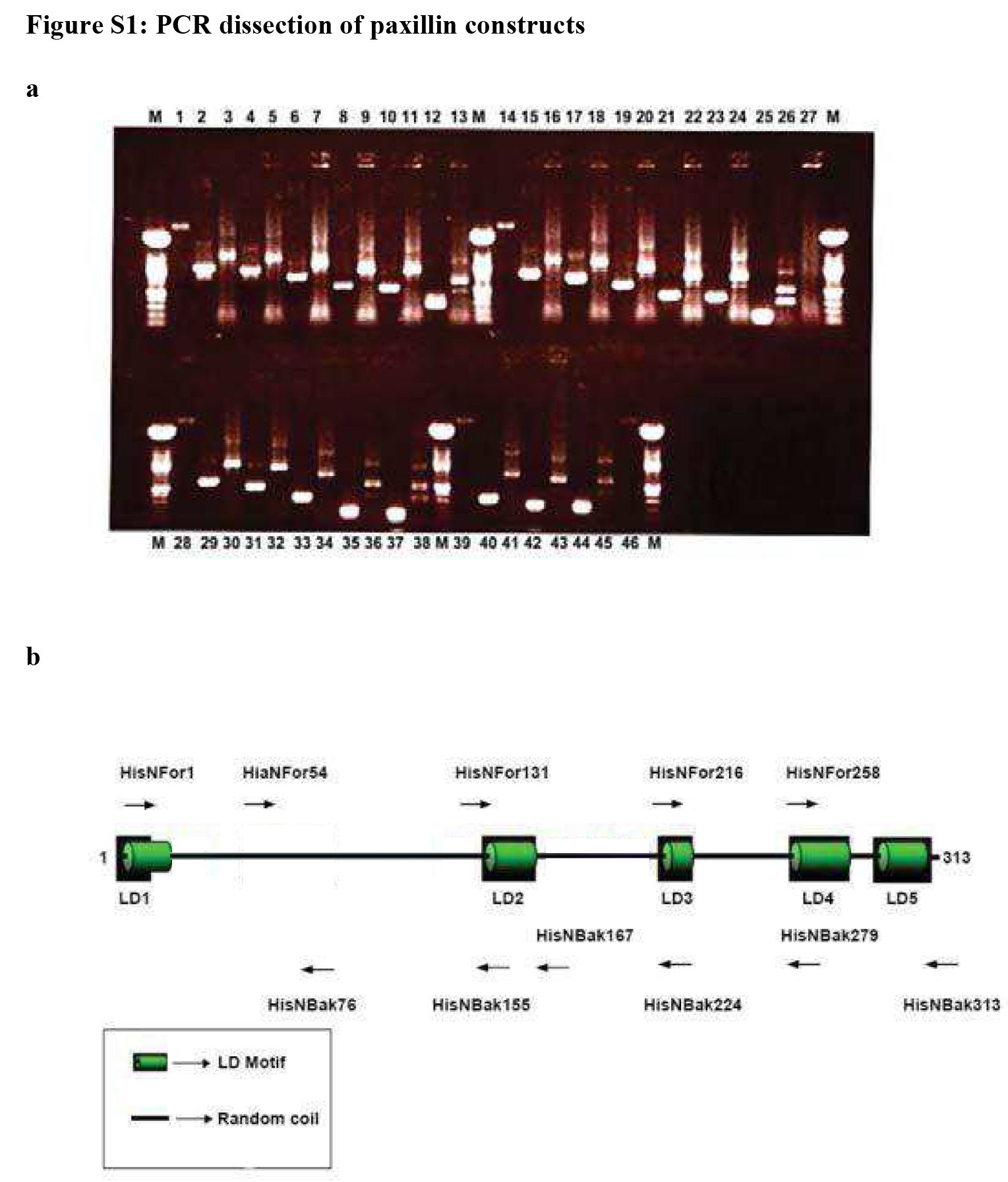

Supplement: S1 Fig — (a): Analysis of primary and secondary PCR products for cell-free protein expression using 0.8% agarose gel. Lanes: M—Markers (100bp); Lanes 2, 4, 6, 8, 10, 12, 15, 17, 19, 21, 23, 25, 29, 31, 33, 35, 37, 40, 42 and 44 show the primary PCR products of constructs A1, A2, A3, A4, A5, A6, B1, B2, B3, B4, B5, B6, C1, C2, C3, C4, C5, D1, D2 and E1, respectively as shown in Fig 1a; Lanes: 1, 14, 27, 28, 39 and 46 are pIVEX2.4d containing T7 promoter (T7P) and T7 terminator (T7T); Lanes 3, 5, 7, 9, 11, 13, 16, 18, 20, 22, 24, 26, 30, 32, 34, 36, 38, 41, 43, and 45 are secondary PCR products obtained from the splicing of primary PCR products and pIVEX2.4d to incorporate the T7P and T7T for obtaining linear DNA templates of constructs A1, A2, A3, A4, A5, A6, B1, B2, B3, B4, B5, B6, C1, C2, C3, C4, C5, D1, D2 and E1 respectively; (b): Schematic of human paxillin (residues 1–313) showing oligonucleotides (forward and reverse primers) used for primary PCR to dissect the molecule. (TIF) [file pone.0150153.s001.tif]

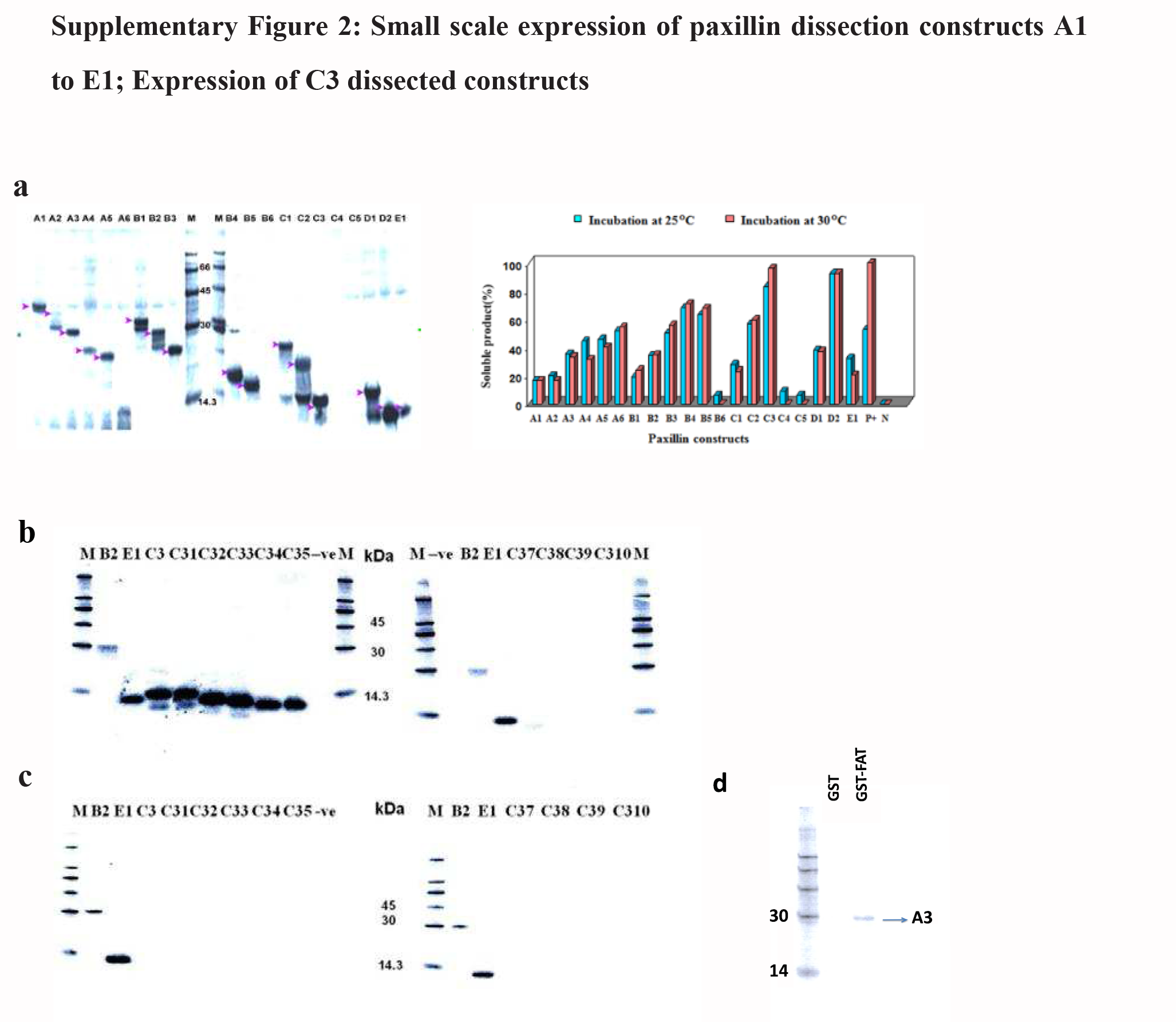

Supplement: S2 Fig — (a): Phosphor screen image of 10% SDS PAGE gel for 35S labeled paxillin constructs (indicated by the purple arrowhead) (left). Optimization of protein expression was performed at 25°C and 30°C with 8.33μg/ml of template DNA, here the positive control was GFP (+ve) expressed the same conditions and negative control (-ve) was cell-free extract without DNA [1]. The right panel shows the extent of solubility of each construct; (b): Phosphor screen image of 10% SDS PAGE gel for 35S labeled, small scale expressed C3 dissected constructs. (c): GST pull down assay showing B2 and E1 interaction and C3 deleted constructs (C31-C35) showing lack of interaction. (d): Interaction assay of a paxillin fragment, A3 showing its non-interaction with GST. (TIF) [file pone.0150153.s002.tif]

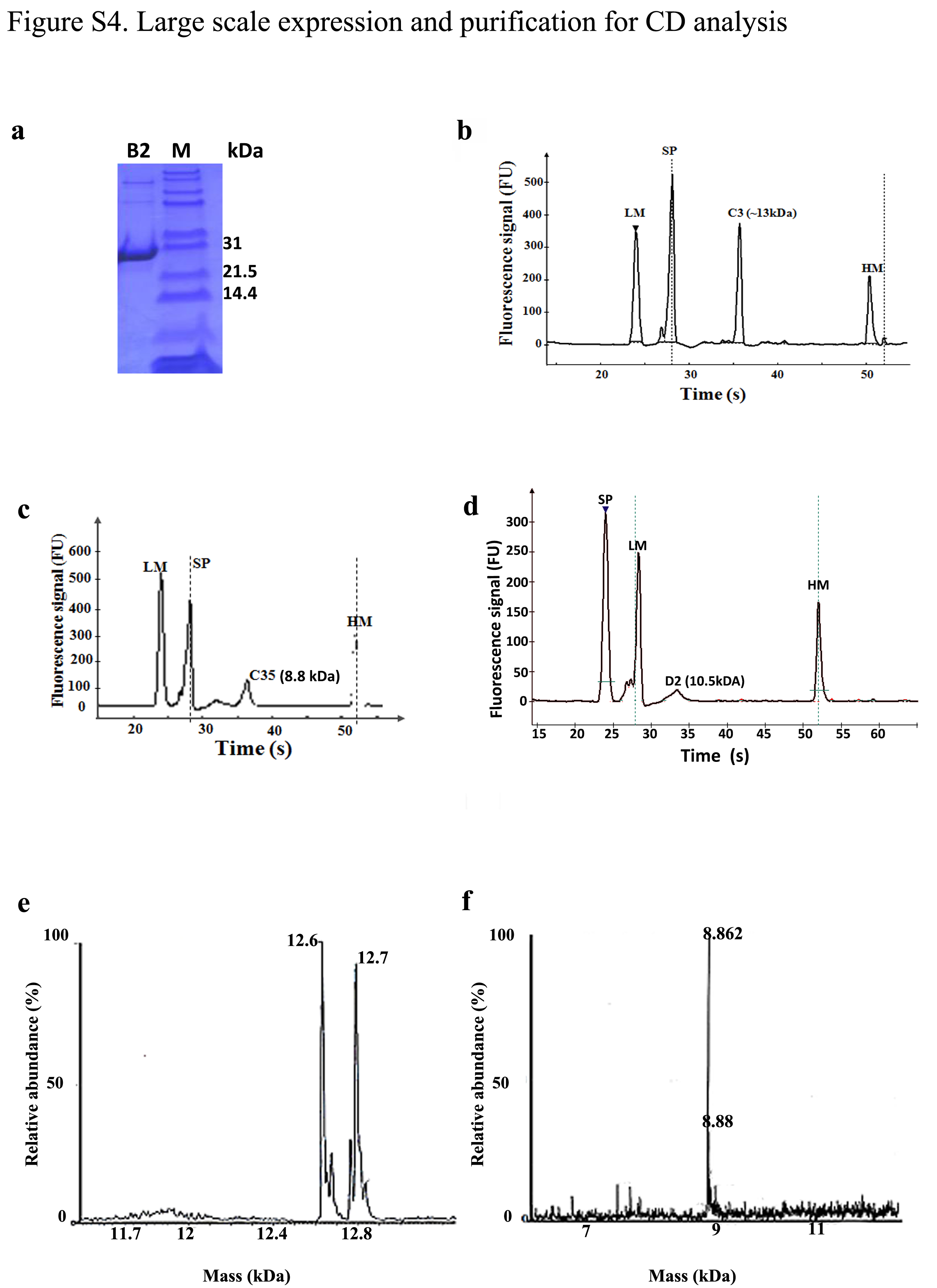

Supplement: S3 Fig — (a): 12% SDS PAGE analysis of expressed and purified B2. M-Marker. The proteins were visualized with Coomassie Brilliant Blue; (b): Capillary electrophoresis (using Agilent Bioanalyser) of paxillin construct C3 under non-reducing conditions. The protein size is estimated by comparison with protein standards (6 to 53 kDa) and the sample concentration by comparison of peak area; (c): Capillary electrophoresis (using Agilent Bioanalyser) of paxillin construct C35 under non-reducing conditions; (d): Capillary electrophoresis (using Agilent Bioanalyser) of paxillin construct D2 under non-reducing conditions. SP: System peak; LM: Lower marker; HM: Higher marker (e): The deconvoluted ESI-mass spectrum of paxillin C3 construct. The calculated MW for the major species agrees with two different molecular weights to the same species of C3. The mass difference between the two main peaks (12792Da -12634Da = 158Da) is the approximate difference expected for deletion of an amino terminal formyl methionine (159Da); (f): The deconvoluted ESI-mass spectrum of paxillin C35 construct. The calculated MW (8862 Da) of the major species exactly agrees with a protein without an amino-terminal methionine, as expressed from pIVEX2.4dC35. (TIF) [file pone.0150153.s003.tif]
