# Supplementary material for: Deciphering Mode of Action of Functionally Important Regions in the Intrinsically Disordered Paxillin (Residues 1-313) Using Its Interaction with FAT (Focal Adhesion Targeting Domain of Focal Adhesion Kinase)
Source: PLoS One. 2016 Feb 29;11(2):e0150153. doi: 10.1371/journal.pone.0150153 (PMC4771712; doi:10.1371/journal.pone.0150153)

Supplementary Fig. 4: Expression and purification of B2, C35, C35_1, C35_2 and C35_3 for Bio-Layer Interferometry studies

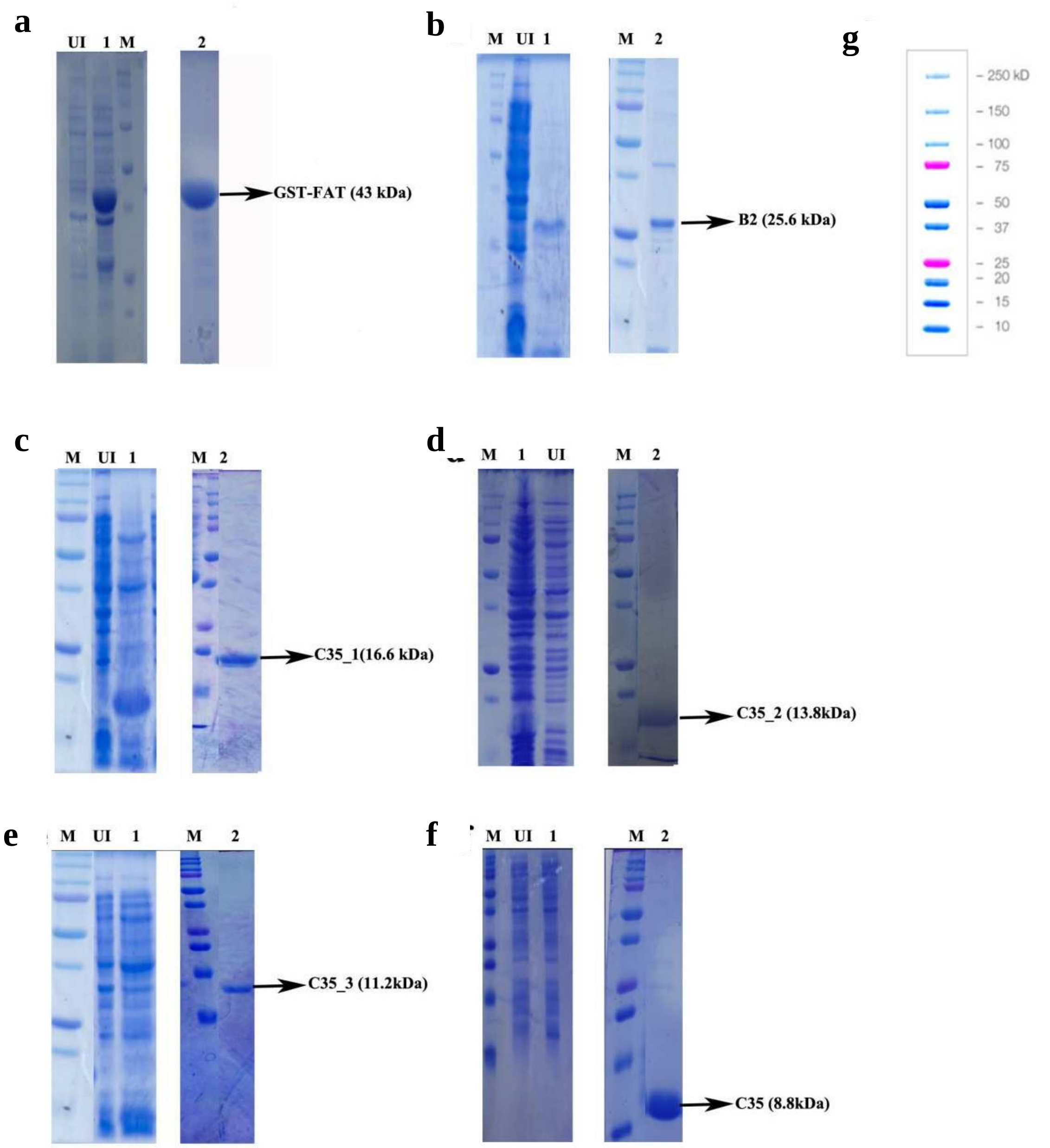

Supplement: S4 Fig — All the above constructs were expressed in E.coli, BL21DE3 strain. UI refers to uninduced and M refers to Marker. GST-FAT, B2 and C35_1 were run on 12% SDS PAGE, C35_2, C35_3 and C35 were run on 15% SDS PAGE. The proteins were visualized with Coomassie Brilliant Blue. (a) Lane 1: Soluble fraction of expressed GST-FAT; Lane 2: Purified GST-FAT. (b) Lane 1: Soluble fraction of B2; Lane 2: Purified fraction of B2. (c) Lane 1: Soluble fraction of C35_1; Lane 2: Purified fraction of C35_1. (d) Lane 1: Soluble fraction of C35_2; Lane 2: Purified fraction of C35_2. (e) Lane 1: Soluble fraction of C35_3; Lane 2: Purified fraction of C35_3. (f) Lane 1: Soluble fraction of C35; Lane 2: Purified fraction of C35. (g) Precession plus dual color Molecular weight marker with kDa values. (PDF) [file pone.0150153.s004.pdf]
