# Supplementary material for: Deciphering Mode of Action of Functionally Important Regions in the Intrinsically Disordered Paxillin (Residues 1-313) Using Its Interaction with FAT (Focal Adhesion Targeting Domain of Focal Adhesion Kinase)
Source: PLoS One. 2016 Feb 29;11(2):e0150153. doi: 10.1371/journal.pone.0150153 (PMC4771712; doi:10.1371/journal.pone.0150153)

Fig 5 Global curve fitting of the curves obtained with Bio-layer interferometry studies, where the paxillin constructs were subjected to interaction with GST-FAT bound to Anti-GST biosensor

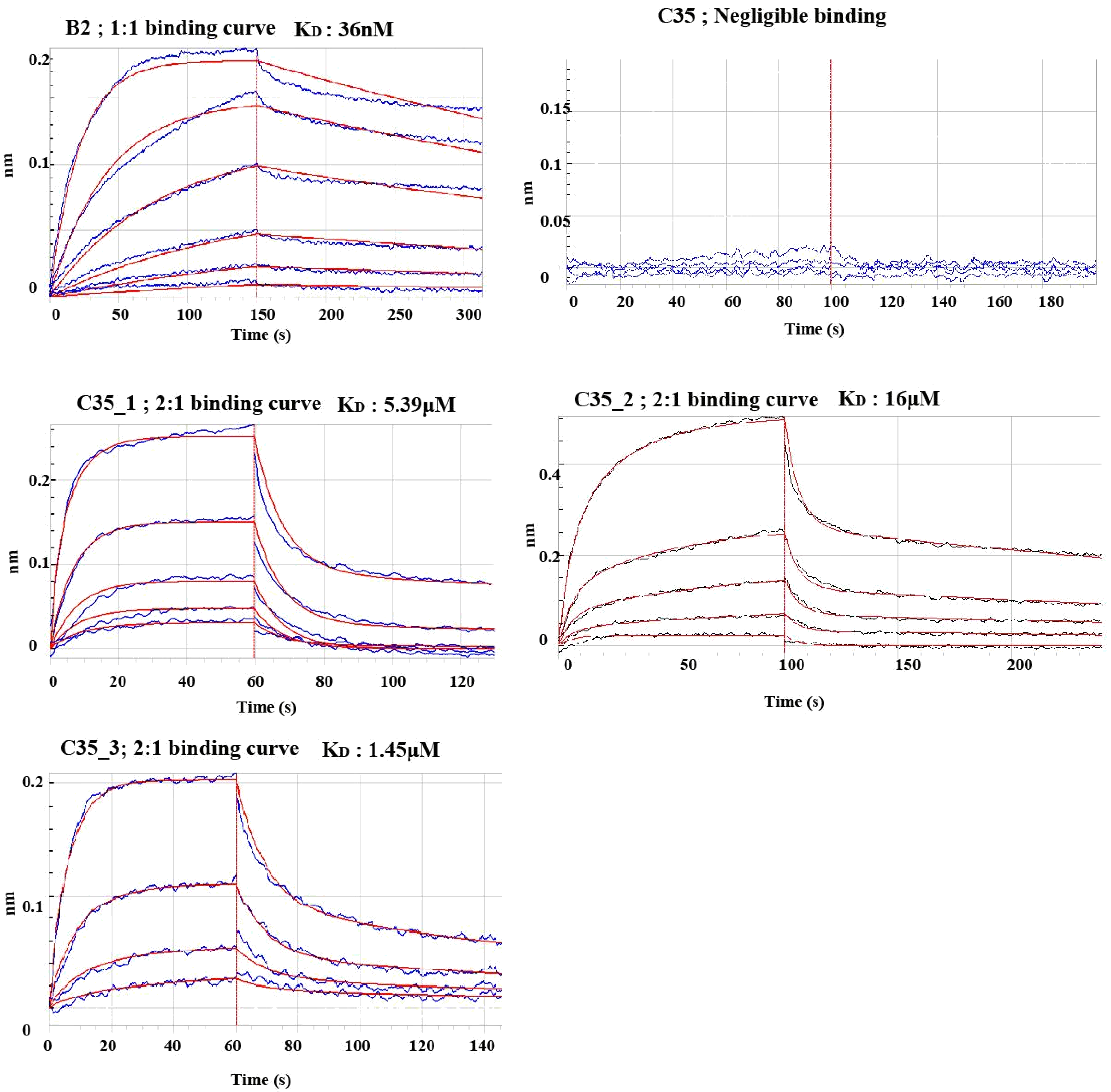

Supplement: S5 Fig — B2 shows maximum binding with KD value in the nano-molar range and the curves fit into a 1:1 binding model. C35 shows negligible binding and the rest of the constructs show binding lower than B2 with KD values in micro-molar range and the curves fit into a 2:1 binding model. (PDF) [file pone.0150153.s005.pdf]
