# Supplementary material for: Deciphering Mode of Action of Functionally Important Regions in the Intrinsically Disordered Paxillin (Residues 1-313) Using Its Interaction with FAT (Focal Adhesion Targeting Domain of Focal Adhesion Kinase)
Source: PLoS One. 2016 Feb 29;11(2):e0150153. doi: 10.1371/journal.pone.0150153 (PMC4771712; doi:10.1371/journal.pone.0150153)
Supplement: S1 Table — (PDF) [file pone.0150153.s006.pdf]

Table S1: Primers used for the generation of linear DNA templates with N-terminal His-tag of dissected constructs of paxillin and primers used for amplification of FAT, C35_1, C35_2, C35_3 and C35 for Bio-layer Interferometry studies

Primer name	Primer sequence
HISNFOR1	CGCTTAATTAAACATATGACCATGGATGATTTAGATGCACTTCTGGCGG
HISNFOR54	CGCTTAATTAAACATATGACCAGCGAGGCCCTCAATGG
HISNFOR131	CGCTTAATTAAACATATGACCACCGTAATGAGCACGTCC
HISNFOR216	CGCTTAATTAAACATATGACCGGTGGAGAGTCTCTTGGAT
HISNFOR258	CGCTTAATTAAACATATGACCTCGGCCTCCTCTGCCAC
HISBAK76	TTAGTTAGTTACCGGATCCCTTAGATGAATCGGGAGCCGCT
HISBAK155	TTAGTTAGTTACCGGATCCCTTATCCAGCTGTACAGCGTTCAGT
HISBAK167	TTAGTTAGTTACCGGATCCCTTAGGGTTGGCCTCATCTGCA
HISBAK224	TTAGTTAGTTACCGGATCCCTTAAAGGCTCTCCAGTTCATCC
HISBAK279	TTAGTTAGTTACCGGATCCCTTATCCGGCCATGAACTTGAAA
HISBAK313	TTAGTTAGTTACCGGATCCCTTACAGGTCAGACTGCAGGCT
HISNFOR131	CGCTTAATTAAACATATGACCACCGTAATGAGCACGTCC
HISNBAK224	TTAGTTAGTTACCGGATCCCTTAGCTCTCCAGTTCATCCAAG
HISNBAK218	TTAGTTAGTTACCGGATCCCTTAGAGACTCTCCACACTGG
HISNBAK210	TTAGTTAGTTACCGGATCCCTTAGTCCTCCAGGCCCC
HISNBAK203	TTAGTTAGTTACCGGATCCCTTAATTCCGCCTAGGCTTCTCT
HISNBAK196	TTAGTTAGTTACCGGATCCCTTACGTCAGGGGCCCAG
HISNBAK189	TTAGTTAGTTACCGGATCCCTTATCCCAAGGGGCTGTTAGTC
HISNBAK182	TTAGTTAGTTACCGGATCCCTTATGGGACACCATAGAGGGG
HISNBAK175	TTAGTTAGTTACCGGATCCCTTACAGGGCCCCAGGAAG
HISNBAK168	TTAGTTAGTTACCGGATCCCTTAGCTTGAGTTGGCCTCATCT
HISNBAK161	TTAGTTAGTTACCGGATCCCTTAAGGGAAGCCTGGTGGG
HISNBAK154	TTAGTTAGTTACCGGATCCCTTACTGTACAGCGTTCAGTTCC
FAT(BamHI)_Forward primer	GAAGGATCCCAGCCCTGCTGACAGCTACAACG
FAT(Not1)_Reverse primer	CGAGACCACACTGAGCGGCCGCAAG
C35(Sal1)_Forward primer	CAATCGCATATGCCCACCGTAATGAGCACGTC
C35 (Not1)_Reverse primer	GCGCGGAATTCTTAGGGGCTGTTAGTCTCTGG
B2(Nde1)_Forward primer	CGCGCCATATGACAATCCTTGACCCCTTAGAC
B2(EcoR1)_Reverse primer	GCGCGGAATTCTTACATGAACTTGAAATCCG
C35_1(Nde1)_Forward primer	CGCGCCATATGTCCAGCGAGGCCCTCAATG
C35_1(EcoR1)_Reverse primer	GCGCGGAATTCTTAGGGGCTGTTAGTCTCTGG
C35_2(Nde1)_Forward primer	CGCGCCATATGCAGCAGCCTCAGTCCTCATCAC
C35_2(EcoR1)_Reverse primer	GCGCGGAATTCTTAGGGGCTGTTAGTCTCTGG
C35_3(Nde1)_Forward primer	CGCGCCATATGGGCTCTCCGTGCTCCCGAGTG

C35_3(EcoR1)_Reverse primer	GCGCGGAATTCTTAGGGGCTGTTAGTCTCTGG

Underlined and non underlined primer sequences in HISFOR1, HISFOR54, HISFOR131, HISFOR216, HISFOR258 and HISNFOR131 are 5’ overlapping sequence of pIVEX2.4d (25) and 5’ overlapping sequence of paxillin, respectively. Underlined and non underlined primer sequences in HISNBAK76, HISNBAK155, HISNBAK167, HISNBAK224, HISNBAK279, HISNBAK313, HISNBAK224, HISNBAK218, HISNBAK210, HISNBAK203, HISNBAK196, HISNBAK189, HISNBAK182, HISNBAK175, HISNBAK168, HISNBAK161 and HISNBAK154 are 3’ overlapping sequence of pIVEX2.4d and 3’overlapping sequence of paxillin, respectively. The primers with names starting with FAT, C35, B2, C35_1, C35_2 and C35_3 are primers used for amplification of FAT, C35_1, C35_2, C35_3 and C35 for bio-layer interferometry studies.
