# Supplementary material for: Deciphering Mode of Action of Functionally Important Regions in the Intrinsically Disordered Paxillin (Residues 1-313) Using Its Interaction with FAT (Focal Adhesion Targeting Domain of Focal Adhesion Kinase)
Source: PLoS One. 2016 Feb 29;11(2):e0150153. doi: 10.1371/journal.pone.0150153 (PMC4771712; doi:10.1371/journal.pone.0150153)
Supplement: S2 Table — (PDF) [file pone.0150153.s007.pdf]

Table S2: Expression of N-terminal Constructs with the incubation temperature

Constructs	Expression (% respect to GFP)	Incubation temperature (C)
A1	6	25
A2	6.7	25
A3	12.6	25
A4	14	25
A5	14	30
A6	23	25
B1	20	25
B2	16	25
B3	26	25
B4	34	30
B5	29	30
B6	4	25
C1	11	30
C2	28	25
C3	39	30
C4	No expression	
C5	<4	30
D1	23	25
D2	41	30
E1	14	25
