# Supplementary material for: Deciphering Mode of Action of Functionally Important Regions in the Intrinsically Disordered Paxillin (Residues 1-313) Using Its Interaction with FAT (Focal Adhesion Targeting Domain of Focal Adhesion Kinase)
Source: PLoS One. 2016 Feb 29;11(2):e0150153. doi: 10.1371/journal.pone.0150153 (PMC4771712; doi:10.1371/journal.pone.0150153)
Supplement: S3 Table — (PDF) [file pone.0150153.s008.pdf]

Table S3 : Alpha helix, Beta helix content and random coil content of constructs B2, C3 and C35 and the LD motifs according to CD analysis

Constructs	α-helix (%)	Random coil (%)	β sheet (%)
B2	12.43	82.37	5.2
C3	37.89	56.97	5.14
C35	95.35	4.55	0.1
LD1	35.3	63.63	1.07
LD2	94.97	5.01	0.02
LD3	17.65	78.59	3.76
LD4	94.65	5.33	0.02
LD5	12.43	82.37	5.2
D2	95.03	4.87	0.1
