# Supplementary material for: Deciphering Mode of Action of Functionally Important Regions in the Intrinsically Disordered Paxillin (Residues 1-313) Using Its Interaction with FAT (Focal Adhesion Targeting Domain of Focal Adhesion Kinase)
Source: PLoS One. 2016 Feb 29;11(2):e0150153. doi: 10.1371/journal.pone.0150153 (PMC4771712; doi:10.1371/journal.pone.0150153)
Supplement: S4 Table — (PDF) [file pone.0150153.s009.pdf]

Table S4: Parameters and details for the paxillin constructs

Paxillin construct	No. of amino acids	Molecular Weight	Theoretical pI	No of Methionines
A1	336	35660.4	5.34	8
A2	305	32667.1	5.33	7
A3	251	26870.6	5.39	4
A4	193	20842.9	5.42	4
A5	181	19645.6	5.57	4
A6	102	11258.6	5.75	3
B1	282	29755.8	5.79	7
B2	251	26762.5	5.79	6
B3	197	20966.0	5.90	3
B4	139	14988.2	6.01	3
B5	127	13741.0	6.28	3
B6	48	5353.9	7.07	2
C1	205	21528.0	6.05	7
C2	174	18534.7	6.04	6
C3	120	12763.2	6.21	3
C4	62	6760.5	6.41	3
C5	50	5513.2	7.07	3
D1	120	12732.1	6.12	6
D2	89	9738.8	6.12	5
E1	79	8310.0	7.07	5
