# Supplementary material for: Deciphering Mode of Action of Functionally Important Regions in the Intrinsically Disordered Paxillin (Residues 1-313) Using Its Interaction with FAT (Focal Adhesion Targeting Domain of Focal Adhesion Kinase)
Source: PLoS One. 2016 Feb 29;11(2):e0150153. doi: 10.1371/journal.pone.0150153 (PMC4771712; doi:10.1371/journal.pone.0150153)
Supplement: S1 Text — (DOCX) [file pone.0150153.s010.docx]

**S1 text: Equations for the calculations of k_on_, k_off_ and K_D_**

The equations used for calculations of k_on_, k_off_ and K_D_ for the 1: 1 binding model are provided below. 2:1 binding model would be a combination of two 1:1 binding models since two analyte molecules bind the ligand sites independently:

1. Association phase:

$y=R_{\max}\frac{1}{1+\frac{k_{off}}{k_{on}*[Analyte]}}(1-e^{-(k_{on}*[Analyte]+k_{off})t})$ (1)

y is the level of binding, R_max_ is the maximum binding signal, k_on_ is the association rate, k_off_ is the dissociation rate, [Analyte] is the concentration of analyte and t is the time.

1. Dissociation phase:

$y=y_{0}e^{-k_{dis}(t-t_{0})}$ (2)

t_0_ is the time at the start of dissociation.

$y_{0}=R_{\max}\frac{1}{1+\frac{k_{off}}{k_{on}*[Analyte]}}(1-e^{-(k_{on}*[Analyte]+k_{off})t_{0}})$ (3)

$K_{D}=\frac{k_{off}}{k_{on}}$ (4)

K_D_ is the equilibrium dissociation constant.
